# The visual geometry of a tool modulates generalization during adaptation

**DOI:** 10.1038/s41598-019-39507-5

**Published:** 2019-02-25

**Authors:** Mohsen Sadeghi, Hannah R. Sheahan, James N. Ingram, Daniel M. Wolpert

**Affiliations:** 1 Department of Engineering, University of Cambridge, Trumpington Street, Cambridge, CB2 1PZ UK; 2 Zuckerman Mind Brain Behavior Institute, Department of Neuroscience, Columbia University, New York, United States

## Abstract

Knowledge about a tool’s dynamics can be acquired from the visual configuration of the tool and through physical interaction. Here, we examine how visual information affects the generalization of dynamic learning during tool use. Subjects rotated a virtual hammer-like object while we varied the object dynamics separately for two rotational directions. This allowed us to quantify the coupling of adaptation between the directions, that is, how adaptation transferred from one direction to the other. Two groups experienced the same dynamics of the object. For one group, the object’s visual configuration was displayed, while for the other, the visual display was uninformative as to the dynamics. We fit a range of context-dependent state-space models to the data, comparing different forms of coupling. We found that when the object’s visual configuration was explicitly provided, there was substantial coupling, such that 31% of learning in one direction transferred to the other. In contrast, when the visual configuration was ambiguous, despite experiencing the same dynamics, the coupling was reduced to 12%. Our results suggest that generalization of dynamic learning of a tool relies, not only on its dynamic behaviour, but also on the visual configuration with which the dynamics is associated.

## Introduction

Successful object manipulation requires the formation of internal models that map between the desired dynamic behaviour of an object and the appropriate motor commands^1–3^. The dynamic properties of objects often can vary with movement contexts, which in turn change over time. For example, when using a handsaw, considerable force is required to execute the down-stroke of the movement, but relatively little for the upstroke. In this case, multiple representations are suggested to be formed for efficient manipulation of a single object under these different contexts^4,5^. Understanding sensorimotor learning in object manipulation requires knowledge of how such representations interact and how they are updated through learning.

Information about the dynamics of an object can be inferred in two main ways—visual cues about the object’s configuration can be attained to build initial predictions of an object’s behaviour, while information can also be acquired through physically interacting with the object. Making accurate inferences over time about the physical behaviour of an object is critical to the representation of its dynamics and successful control. Studies have shown that viewing an object’s size or shape, or observing static images of an object’s interaction with the external world, strongly informs inferences about that object’s dynamic properties, prior to attempts at control^6–10^. For example, given the visual configuration of an object on a surface, subjects can accurately predict its stability or locate its centre of mass^6,7,11^. Moreover, it has been suggested that visual information rapidly informs appropriate predictive control strategies when objects are lifted^12–19^ or rotated^4^. However, the extent to which visual information contributes to the formation or interaction of context-dependent representations is poorly understood.

Here we examine how providing or withholding visual information about the configuration of an object affects the representation of its dynamics during adaptation, and how this representation generalizes from one context to another. Subjects performed reciprocal, back-and-forth rotations of a virtual hammer-like object in a horizontal plane. The dynamics of the object could vary for each rotational direction, allowing us to examine the coupling of adaptation, that is, how adaptation in one direction is transferred to the adaptive behaviour for rotations in the other direction. We manipulated the visual configuration of the object by either providing an explicit visual configuration (i.e., the shape of a hammer) or an ambiguous configuration that was not informative as to the object dynamics. We asked how the visual configuration of the object modulated the coupling of adaptation between back and forth rotations. A context-dependent state-space model was developed to quantify coupling in each condition. We found that, despite experiencing the same object dynamics in each condition, the coupling of adaptation was significantly enhanced when the visual configuration of the object was explicitly provided. This suggests that an explicit visual configuration, which is informative of the object dynamics, contributes to the formation of a more cohesive dynamic representation across contexts (rotational directions).

## Results

**Figure 1:**
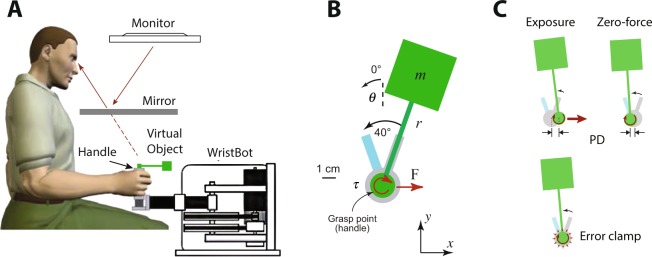
Experimental setup. (**A**) Subjects grasped the handle of the WristBot and rotated a virtual hammer-like object back and forth between two angular targets (two rectangular bars separated by 40°). (**B**) The object consisted of a mass (*m*) on the end of a rigid rod (length *r*). The mass of the object was set to 1% of the subject’s body mass. Subjects grasped the object by a handle at the other end of the rod and rotated the object while trying to prevent translational movement of the handle. The dynamics of the object consisted of a torque (*τ*) due to the moment of inertia as well as translational forces (F) due to the circular motion of the mass that perturbed the handle of the object^21^. (**C**) Three types of trials were used. Subjects experienced the torque on all trials. On exposure trials subjects experienced translational forces. On zero-force trials, the handle was free to move. The kinematic error (KE) of the handle during the rotation was measured on exposure and zero-force trials. On error-clamp trials, the handle was fixed in place using a simulated spring that prevented translational movement. On these trials we measured the compensatory forces.

Subjects sat at a virtual reality system and grasped the vertical handle of a robotic manipulandum (the WristBOT^20^; Fig. 1A). They performed reciprocal, back and forth rotations of a virtual hammer-like object by rotating the handle around its vertical axis. The WristBot allowed translational and rotational movements of the handle and could generate forces in the horizontal plane, as well as torques at the handle.

The task required the subject to rotate the object clockwise (CW) and counter-clockwise (CCW) between two angular targets (rectangles, 40° apart; Fig. 1B) while attempting to keep the handle stationary. The robotic manipulandum could simulate the torques and forces associated with rotating the object^4^. We manipulated the dynamics of the object by simulating three different trial-types (Fig. 1C). On all these trial types the torques associated with rotating the object (due to inertia) were always generated. On exposure trials, the full dynamics of the object (torques and forces) were applied (Fig. 1B). On zero-force trials, no forces were applied so that the handle was free to translate. On error-clamp trials, the translation of the handle was clamped by a simulated spring. In each pair of clockwise (CW) and counter-clockwise (CCW) rotations, the dynamics of the object (i.e., trial type) could be separately manipulated for the first rotation (Out) and the second rotation (Return) of a pair. The direction of Out and Return rotations (CW or CCW) was counterbalanced across subjects.

On exposure trials, in order to prevent translation of the handle (as required by the task), subjects had to generate compensatory forces to oppose the translational forces associated with the dynamics of the object^4^. To evaluate performance, we focus on two behavioural measures. On exposure and zero-force trials, we calculated the displacement of the handle from the starting position as a measure of kinematic error (KE). On error-clamp trials, we calculated the regression coefficient (without intercept) between the magnitude of the force generated by the subject against the clamp, and the ideal force magnitude that would fully compensate for the translational forces due to object rotation. This measure represents the adaptation index that directly reflects how well the dynamics of the object is learned (1 for full learning, and 0 for no learning).

**Figure 2:**
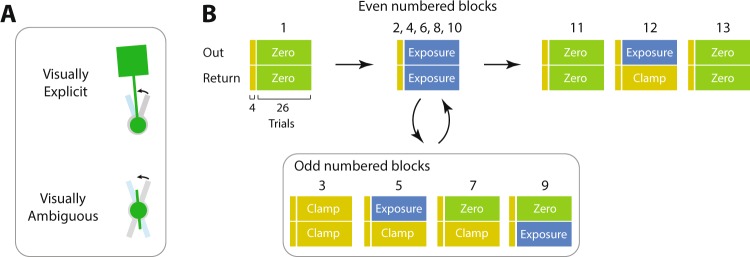
Experimental paradigm. (**A**) In the visually explicit group, the visual information about the object was available and informative of the object’s dynamics. In the visually ambiguous group, the rod and mass were never displayed, and thus the visual geometry of the object was not informative of its dynamics. (**B**) Subjects in both groups performed the same sequence of trials in 13 blocks. Each subject started with a block of zero-force trials (green), followed by 9 blocks which alternated between concurrent exposure (blue blocks) for both Out and Return rotations (even numbered blocks) and blocks in which the dynamics could be varied independently for the Out and Return rotations (odd numbered blocks; dynamics as shown). Subjects then performed a final three blocks as shown. Each block was preceded by 4 error-clamp trials for both the Out and Return rotations (narrow yellow rectangles) so as to probe the adaptation index.

Two groups of subjects (n = 12 in each group) participated in the experiment. Both groups experienced the same trial sequence and object dynamics. The visual configuration of the object, however, differed between the groups. In the first group (visually Explicit group), the visual object consisted of a circular handle attached by a rod to a square mass (Fig. 2A, top). The visual geometry of the object in this group was, therefore, informative of the dynamics. In the second group (visually Ambiguous group), the rod and mass of the object were not displayed (Fig. 2A, bottom). In this case, the visual geometry of the object was not informative of the dynamics.

The experiment consisted of reciprocal Out and Return rotations performed in 13 blocks (Fig. 2B). These blocks were designed to allow us to assess coupling of learning, that is, how errors in one rotational direction (e.g., Out) affect adaptation in the other direction (e.g., Return). Subjects started with a block of zero-force trials as baseline (block 1), followed by 9 blocks which alternated between blocks of concurrent exposure for both Out and Return rotations (the even numbered blocks), and blocks in which the dynamics of the object could vary between the Out and Return rotations (the odd numbered blocks; Fig. 2B). We assessed the adaptation during the concurrent exposure blocks, and examined how such adaptation was coupled between Out and Return rotations when the dynamics differed for each rotation direction (i.e., in odd numbered blocks). Further, in blocks 11 and 13 both rotations were zero-force trials in order to examine the coupling during deadaptation, while block 12 consisted of exposure on Out rotations and error-clamp on the Return rotations. This was used to assess how adapting to the Out rotation from baseline influenced adaptation on the Return rotation.

**Figure 3:**
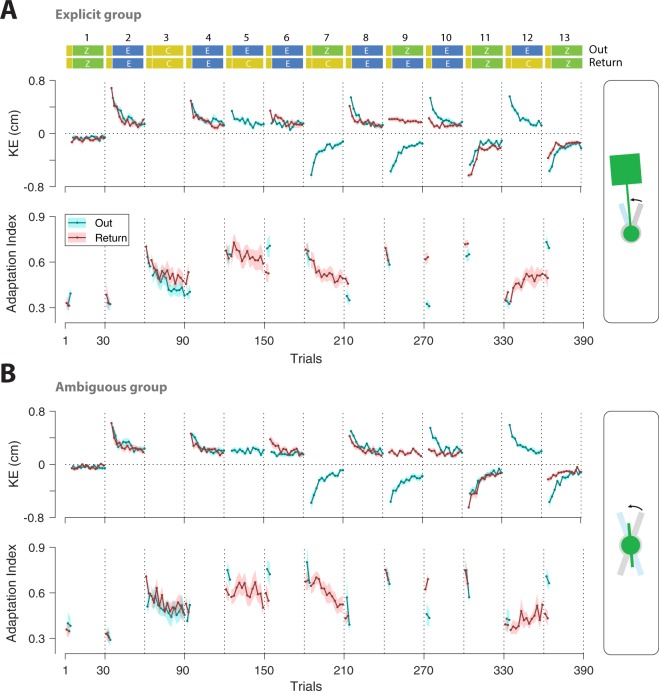
Experimental results. The KE (exposure and zero-force trials) and the adaptation index (error-clamp trials) for the (**A**) Visually Explicit and (**B**) Visually Ambiguous groups. Data is plotted separately for Out and Return trials and shows mean ± s.e. (shading) across subjects.

Figure 3 illustrates the time series of kinematic error (KE; exposure and zero-force trials) and adaptation index (clamp trials) for Out (cyan) and Return (red) movements for both groups. Consistent with previous studies^4,21^ subjects rapidly adapted/re-adapted to the changing dynamics of the object during exposure blocks (e.g., rapid reduction of KE on even-numbered blocks 2–10). The final level of KE in the early exposure blocks was different between the Ambiguous and the Explicit group: the final level of KE (last 10 trials, combined for Out and Return movements) for block 2 (first exposure block) was larger for the Ambiguous group (mean ± s.e., 0.23 ± 0.03) compared to the Explicit group (mean ± s.e., 0.15 ± 0.017; one-way ANOVA: ${F}_{1,22}=6.00$, $p=0.023$). Such a difference, which is also observed in previous studies^22^, likely arises from the lack of visual information about the object’s configuration in the Ambiguous group, which leads to a larger KE for this group. In the later exposure blocks, however, this difference is attenuated as learning progresses (the mean ± s.e. of final KE over blocks 4–10: 0.184 ± 0.02 for the Ambiguous, and 0.14 ± 0.019 for the Explicit group; one-way ANOVA: ${F}_{1,22}=2.58$, $p=0.123$).

The coupling of adaptation between Out and Return movements was assessed by examining blocks in which the object dynamics differed between the rotations. For example, coupling would be reflected in different retention time series on error-clamp trials for the Return movements in blocks 5 and 7 in which the Out movements were Exposure and Zero-force trials, respectively. Similarly, after deadaptation, coupling would lead to adaptation on error-clamp Return trials of block 12, in which the Out movements are Exposure trials. We first perform a model-free analysis of the adaptation in these key blocks. We follow this with a model-based analysis to quantify coupling.

**Figure 4:**
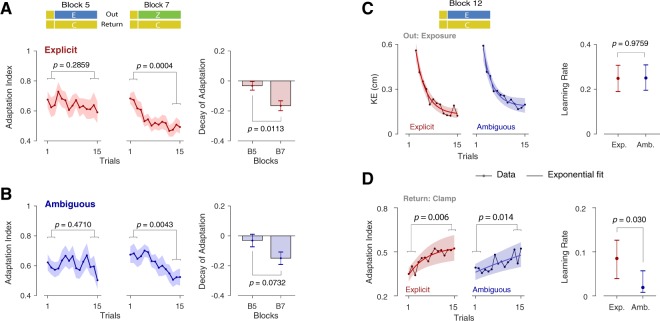
Analysis of coupling across specific blocks. The adaptation index for the error-clamped Return trials of Block 5 and 7 (B5 and B7), shown for the (**A**) Explicit and (**B**) Ambiguous groups. In each block, the initial and final level of adaptation index are compared, and the difference is represented as the amount of adaptation decay in that block (bar plots mean ± s.e. across subjects). (**C**) The KE data and the corresponding single-rate state space model fits for the Out movements (exposure trials) of block 12, for Explicit (Exp.) and Ambiguous (Amb.) groups. (**D**) The adaptation index and the fits for the error-clamped Return trials in block 12. The shading on the fits and the error bars on the learning rate represent the 95% confidence intervals from bootstrapping (see Methods). The *p*-values for the difference between the parameters are obtained from the bootstrap (two-tailed test).

Figure 4 shows the retention of adaptation on the Return trials of block 5 and 7 for the Explicit (Fig. 4A) and Ambiguous (Fig. 4B) groups. Coupling would be expected to lead to greater retention on block 5 (Out exposure trials) compared to block 7 (Out zero-force trials). To examine any difference in retention, we compared the initial and final level of adaptation index (first and last three error-clamp trials) on the Return movements in each block. The levels of adaptation index for block 5 were not significantly different (Fig. 4A and B; paired t-test: t(11) = 1.122, *p* = 0.286 for the Explicit, and t(11) = 0.747, *p* = 0.471 for the Ambiguous group), indicating protection of adaptation in the block. In contrast, for block 7, the adaptation index was significantly reduced (t(11) = 5.048, $p < 0.001$ for the Explicit, and t(11) = 3.587, *p* = 0.004 for the Ambiguous group). The difference between the initial and final adaptation in each block represents the decay of adaptation, which was larger for block 7 compared to block 5 for both groups, with a significant effect observed for the Explicit group (t(11) = 3.035, *p* = 0.011 for the Explicit and t(11) = 1.98, *p* = 0.073 for the Ambiguous group; Fig. 4A and B, rightmost panel). The amount of decay in each block, however, was similar across groups (one-way ANOVA: ${F}_{1,22}=0.001$, $p=0.98$ for block 5 and ${F}_{1,22}=0.087$, $p=0.77$ for block 7). These results suggest that the decay of adaptation could reflect coupling for both groups, although it could not differentiate the extent of coupling between groups.

We also examined adaptation in block 12, where, after washout, subjects experienced exposure trials on the Out movements while the Return movements were error-clamped. Both groups showed adaptation for the Out movements (reduction of KE; Fig. 4C), while also showing a significant increase in adaptation index for the Return movements (Fig. 4D; the adaptation index in the final three trials of the block was significantly larger than the adaptation in the initial three trials for both the Explicit, $t(11)=3.44$, $p=0.006$, and the Ambiguous, $t(11)=2.93$, $p=0.014$ groups). Due to rapid learning, the initial and final level of KE (Out) and adaptation index (Return) were similar across groups. In particular, the mean ± s.e. of increase in adaptation index (i.e., difference between the initial and final three trials) for the Return movements was not significantly different between the Explicit (0.15 ± 0.044) and the Ambiguous groups (0.11 ± 0.036; one-way ANOVA: ${F}_{1,22}=0.597$, $p=0.448$). This suggested that by the end of the block, the level of adaptation was similar for both groups. However, the rate of increase in adaptation, that is, how fast adaptation had reached the final level, differed across groups significantly (see below). In order to better quantify the rate of learning, for each group we fit a simple single-rate state-space model (see Methods) to the KE (Out movement) and adaptation index (Return movement) of block 12, separately. The learning rate obtained for each performance measure (KE or adaptation index) was then compared across groups. As shown in Fig. 4C, despite similar learning on the outward movement across both groups (two-tailed test on bootstrap distributions, *p* = 0.976; see Methods) the Ambiguous group showed significantly slower learning on the Return movement (two-tailed test for learning rate difference, *p* = 0.03; Fig. 4D). As such, although the final level of adaptation index for Return trials was similar across groups, this level was achieved faster for the Explicit compared to the Ambiguous group. This suggests that the per-trial transfer of learning from Out to Return movements (i.e., coupling) may be different across groups, and hence be modulated by the nature of the visual information provided about the object (i.e., explicit or ambiguous). To quantify such coupling, a thorough model-based analysis is performed.

## Coupling Model

A state-space model was used to evaluate the coupling between Out and Return rotations. The model includes separate states for Out and Return movements and potentially two different rates of adaptation for each^23–25^. The full model, therefore, has 4 states (two rotational directions and two rates) which we represent as a state vector:

1 $${\bf{x}}=[{x}_{{\rm{o}}}^{1}\,{x}_{{\rm{o}}}^{2}\,{x}_{{\rm{r}}}^{1}\,{x}_{{\rm{r}}}^{2}],$$

where subscripts represent the state associated with the movement direction (o = Out and r = Return) and superscripts represent the two rates (1 and 2, e.g. fast and slow). The motor output *y*(*n*) on trial *n* is the sum of the two states associated with the given movement direction, and is determined by the binary elements of the selection vector **q**(*n*):

2 $$y(n)={\bf{q}}(n)\cdot {\bf{x}}{(n)}^{{\rm{T}}}$$

where ${\bf{q}}(n)=[1,1,0,0]$ for Out movements and $[0,0,1,1]$ for Return movements. The error *e*(*n*) on each trial is the difference between the motor output and the task perturbation:

3 $$e(n)=f(n)-y(n)$$

where, *f*(*n*) represents the external perturbation (object force) and is either 1 (for exposure trials) or 0 (for zero-force trials). For channel trials, the error is set to 0 by definition. After each trial, the state vector is updated as follows:

4 $${\bf{x}}(n+1)={\bf{A}}\odot {\bf{x}}(n)+{\bf{B}}\odot {\bf{C}}\cdot e(n)$$

where $\odot$ is element-wise multiplication and ${\bf{A}}=[{\alpha }^{1},{\alpha }^{2},{\alpha }^{1},{\alpha }^{2}]$ and ${\bf{B}}=[{\beta }^{1},{\beta }^{2},{\beta }^{1},{\beta }^{2}]$ represent, respectively, the retention factors and the learning rates. **C** represents a coupling vector that determines how much of the error for one direction of rotation should update the states associated with the other direction of rotation. Therefore this term can couple the learning between the Out and Return movements. Specifically, ${\bf{C}}=[1,\,1,\,{c}^{1},\,{c}^{2}]$ and $[{c}^{1},\,{c}^{2},\,1,\,1]$ for Out and Return movements, respectively, where *c*^1^ and *c*^2^ are the coupling factors for the two rates. Accordingly, on an Out trial, the adaptive states associated with the Out process are fully updated (with a weight of 1), whereas the adaptation states associated with the Return process are updated via the coupling factors *c*^1^ and *c*^2^. The coupling factor can take values between 0 and 1, with 0 as no coupling (Out and Return movements are totally independent) and 1 as full coupling (on each trial Out and Return movements are updated equally based on the error).

The model has 6 parameters that include four rate parameters (two retention factors *α*^1^ and *α*^2^, and two learning rates *β*^1^ and *β*^2^) as well as two coupling factors (*c*^1^ and *c*^2^). Note that we do not constrain the relative values of the rate parameters to exclusively represent fast or slow processes^23^, but rather interpret these values from the fits as being associated with either fast or slow process.

We fit the model to the KE and adaptation index from both groups simultaneously. In this case, the rate parameters were shared between the two groups (same rate parameters were fit to both groups) but separate coupling factors were considered for each group. We could, therefore, directly assess how each condition (visually explicit or visually ambiguous) affects the coupling of adaptation. Specifically, we consider the coupling factors ${c}_{E}^{1}$ and ${c}_{E}^{2}$ to represent the coupling factors for the Explicit group, and ${c}_{A}^{1}$, and ${c}_{A}^{1}$ to account for coupling for the Ambiguous group. The full model, therefore, consists of 8 degrees of freedom (DOF) including four rate parameters (shared between both groups) and four coupling factors (separate for each group).

In addition, we examined several reduced versions of the full model in which we vary the number of states for each direction (single-rate versus dual-rate) as well as the way coupling factors for each group were specified. Specifically, two main sets of models were tested (Table 1). In the first set, we examined models that assumed the same coupling for both Explicit and Ambiguous groups, so that there was no effect of the object’s visual configuration on the coupling (null hypothesis). This consisted of single-rate models (i.e., only *α*^1^ and *β*^1^ as rate parameters while ${\alpha }^{2}={\beta }^{2}=0$) with either full coupling (${c}^{1}=1$; M1), zero coupling (${c}^{1}=0$; M2), or coupling as free parameter (${c}^{1}={k}_{1}$; M3). We also examined dual-rate models with full (${c}^{1,2}=1$; M4), or zero (${c}^{1,2}=0$; M5) coupling for both rates, as well as models in which coupling was fit for the first rate (${c}^{1}={k}_{1}$) while for the second rate it was either full (${c}^{2}=1$; M6), zero (${c}^{2}=0$; M7), same as the first rate (${c}^{2}={c}^{1}$; M8) or could be different from the first rate (${c}^{2}={k}_{2}$; M9).

**Table 1:** Model comparison.

Same coupling for both groups									
Model	Rate parameters	*c* ^1^	*c* ^2^			DOF	ΔBIC	R^2^	% Best fit
M1	*α*^1^, *β*^1^	1	—			2	904	0.8895	0
M2		0	—			2	554	0.9272	0
M3		*k*_1_	—			3	351	0.9433	0
M4	*α*^1^, *β*^1^, *α*^2^, *β*^2^	1	1			4	846	0.8985	0
M5		0	0			4	177	0.9543	0
M6		*k*_1_	1			5	95	0.9588	0
M7		*k*_1_	0			5	49	0.9610	0
M8		*k*_1_	*k*_1_			5	31	0.9619	4.1
M9		*k*_1_	*k*_2_			6	37.4	0.9619	0
**Different coupling for each group**									
**Model**	**Rate parameters**	${{\boldsymbol{c}}}_{{\boldsymbol{E}}}^{{\bf{1}}}$	${{\boldsymbol{c}}}_{{\boldsymbol{A}}}^{{\bf{1}}}$	${{\boldsymbol{c}}}_{{\boldsymbol{E}}}^{{\bf{2}}}$	${{\boldsymbol{c}}}_{{\boldsymbol{A}}}^{{\bf{2}}}$	**DOF**	**ΔBIC**	**R** ^**2**^	**% Best fit**
M10	*α*^1^, *β*^1^	*k*_1_	*k*_2_	—	—	4	336	0.9447	0
M11	*α*^1^, *β*^1^, *α*^2^, *β*^2^	*k*_1_	*k*_2_	1	1	6	64	0.9606	0
M12		*k*_1_	*k*_2_	0	0	6	13.5	0.9629	5.1
M13		*k*_1_	*k*_2_	*k*_1_	*k*_2_	6	0	0.9635	57.7
M14		*k*_1_	*k*_2_	*k*_3_	*k*_3_	7	3.12	0.9637	22.6
M15		*k*_1_	*k*_2_	*k*_3_	*k*_4_	8	6.98	0.9638	10.5

Model comparison between different coupling models. Models varied in their rate parameters (single or dual rate) and in whether their coupling was the same (M1-M9) or could differ (M10-M15) across the Explicit (subscript *E*) and Ambiguous (subscript *A*) groups. In addition, different forms of coupling within each group were considered. The models were all reduced versions (degrees of freedom, DOF) of the full model (M15). Model comparison was performed using the Bayesian Information Criterion (*BIC*; see Methods). The DOF do not include the four mapping parameters which were common across all models and which do not affect the Δ*BIC*s or *R*^2^. The Δ*BIC* represents the difference between the *BIC* of each model and the preferred model with the lowest *BIC* (M13). Note that although the *R*^2^ values might be similar across some models, this measure, unlike *BIC*, is independent of the number of data points and the DOF of the model. Therefore, small improvements in *R*^2^ for a model can translate into a large difference in the likelihood and hence *BIC*. Finally, the ‘% Best fit’ represents the percentage of bootstrap samples (see Methods) in which a given model showed the best performance in terms of *BIC*.

In the second set of models, we allowed different coupling factors for each group. Again we considered a single-rate model (M10) as well as several dual-rate models, in which the first rate had separate coupling for each group (that is, ${c}_{E}^{1}={k}_{1}$ and ${c}_{A}^{1}={k}_{2}$). For the second rate, however, the coupling was either full (${c}_{E,A}^{2}=1$; M11) or zero (${c}_{E,A}^{2}=0$; M12) for both groups, or it was the same as the first rate for each group (${c}_{E/A}^{2}={c}_{E/A}^{1}$; M13), or different from the first rate but same across groups (${c}_{E,A}^{2}={k}_{3}$; M14). Finally, the full version of the model consisted of separate coupling for each rate and group as described above (M15).

To evaluate the coupling, we fit all the models to the average KE and adaptation index from both groups simultaneously. The quality of fit was compared across models based on the Bayesian Information Criterion (*BIC*; see Methods) calculated for each model across the data from both groups. Table 1 shows the *R*^2^ and the relative value of *BIC* (i.e., Δ*BIC*) for each model. The Δ*BIC* represents the difference between the *BIC* of each model, and the *BIC* of the preferred model (M13).

The model comparison can be performed from two different perspectives. First, by comparing the single-rate versus dual-rate models, we see that the fitting quality was substantially poorer for single-rate models with the Δ*BIC* of more than 336 compared to the best dual rate model. This indicates that a single-rate representation of adaptation is insufficient to account for the behavior when the dynamics of the object varies independently for Out and Return rotations.

More importantly, we could compare models that had either the same or different coupling for each group. Models that had separate coupling for each group showed a better fit to the data (i.e., in terms of Δ*BIC*) compared to models with the same coupling for both groups. In particular, model M13 showed the best overall performance with the smallest *BIC*, and model M14 was not far behind. Both models considered separate coupling for each group, indicating a modulation of coupling by the visual information about the object’s configuration. Note that although the *R*^2^ measure for these models might be very close to some models that feature same coupling for both groups (e.g., M8), this measure, unlike *BIC*, is independent of the number of data points and the degrees of freedom (DOF) of the model. Therefore, small improvements in *R*^2^ for a model can translate into a large difference in the likelihood and hence the *BIC*. To further investigate the hypothesis that separate coupling is required for each group (against the null hypothesis that coupling is the same for both groups) we found the percentage of bootstrap samples in which a given model showed the best performance among others in terms of *BIC*. As shown in Table 1 (% Best fit), models that featured separate coupling for each group showed a better performance in more than 95% of the samples. This suggests that the coupling of adaptation in each group is substantially affected by the visual information about the object’s configuration.

**Figure 5:**
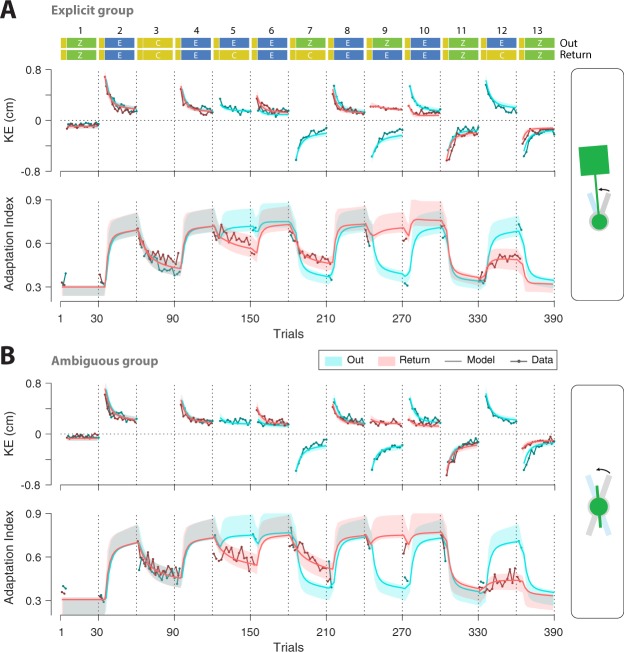
Model fits. The model fits based on the preferred model (M13) for KE and adaptation index for (**A**) Explicit and (**B**) Ambiguous groups. The error bars for the model represent the 95% confidence intervals from bootstrapping.

The way coupling is affected by such explicit or ambiguous visual information could be further examined by looking at the models that best describe the data. In this case, model M13 had the smallest *BIC*, and was the preferred model for more than 57% of the bootstrap samples (Table 1). According to this model, both fast and slow processes (superscripts 1 and 2, Table 1) are equally involved in coupling within each group (i.e., ${c}^{1}={c}^{2}$ for each group), but the extent of such coupling differs across groups. Table 2 shows the best fit parameters for M13 with a coupling of 0.31 for the Explicit group and 0.12 for the Ambiguous group. Figure 5 also illustrates the fits for M13 with the 95% confidence intervals over bootstrap samples (see Methods). A second possible way in which coupling might differ across groups might be based on model M14 which was the preferred model for 22% of the bootstrap samples (i.e., according to % Best fit in Table 1). In this model, the group effect (Explicit or Ambiguous) influences the coupling differently for fast and slow processes, such that for one process, coupling is unaffected by the explicit or ambiguous visual information (i.e., coupling is the same for both groups; ${c}_{E}^{2}={c}_{A}^{2}$; Table 1), while for the other process, it differs across groups (${c}_{E}^{1}\ne {c}_{A}^{1}$). The best fit parameters for M14 revealed that coupling differed across groups via the fast process (i.e., *c*^1^ was associated with the smaller retention factor, *α*^1^ and the larger learning rate *β*^1^), while the slow process shared the same coupling for both groups (that is, the shared coupling *c*^2^ was associated with the larger retention factor *α*^2^, and the smaller learning rate *β*^2^; Table 2).

**Table 2:** Model parameters.

**Model M13**						
*α* ^1^	*α* ^2^	*β* ^1^	*β* ^2^	${c}_{E}^{1}={c}_{E}^{2}$	${c}_{A}^{1}={c}_{A}^{2}$	—
0.8794	0.9897	0.1891	0.0453	0.3070	0.1193	—
**Model M14**						
*α* ^1^	*α* ^2^	*β* ^1^	*β* ^2^	${c}_{E}^{1}$	${c}_{A}^{1}$	${c}_{E}^{2}={c}_{A}^{2}$
0.8809	0.9898	0.1873	0.0445	0.3546	0.0678	0.2146

The best fit parameter values for M13 and M14. In M13, the coupling factor equally affects the fast and slow processes (the superscripts 1 and 2, respectively) and is separately fit to the Explicit (subscript E) and Ambiguous (subscript A) groups. In M14, coupling is separate for each group for the fast process (superscript 1), while shared between the groups for the slow process (superscript 2).

**Figure 6:**
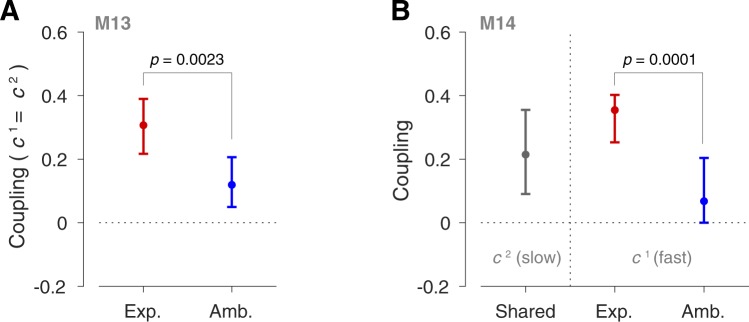
Coupling was enhanced when the visual geometry of the object was explicitly provided. The best fit value of the coupling factor for Explicit (Exp.) and Ambiguous (Amb.) groups based on (**A**) model M13 and (**B**) model M14. The circular markers show the coupling value from fitting the model to the average data across subjects. The error bars represent the 95% confidence intervals of the coupling distribution across bootstrap samples (see Methods). The *p*-value is also obtained from the two-tailed test on the bootstrap distributions.

In Fig. 6 the value of coupling and its confidence intervals across bootstrap samples is compared between Explicit and Ambiguous groups for both M13 and M14 as the most likely models in explaining the data. As shown in Fig. 6A, for M13, a significant amount of coupling is observed for both groups (two-tailed test on the bootstrap distributions: $p < 0.001$ for Explicit and $p=0.004$ for Ambiguous groups), indicating a significant transfer of adaptation from one rotational direction (e.g., Out) to the other (e.g., Return). This is consistent with our earlier analysis of adaptation in the Return movements (Blocks 5, 7 and 12; Fig. 4) showing that coupling exists for both groups (e.g., adaptation decay in Return movements was correlated with the adaptive behaviour in the Out movements; Fig. 4A and B). The extent of this coupling, however, differs significantly between the two groups, with larger coupling for the Explicit group and smaller coupling for the Ambiguous group (two-tailed test on the bootstrap samples: *p* = 0.0023). Similarly for M14 (Fig. 6B), both groups show a significant coupling for the slow process (two-tailed test on the bootstrap samples for the shared coupling: *p* = 0.0034), meaning that coupling exists for both groups. The extent of coupling for the fast process, however, was significantly larger for the Explicit group compared to the Ambiguous group (two-tailed test over bootstrap samples on the coupling difference between groups: *p* = 0.0001).

Overall, the modelling results suggest that coupling exists for both Explicit and Ambiguous conditions, while the extent of coupling is significantly increased when the visual information about the object’s configuration is provided. How the fast and slow processes are involved in such modulation of coupling is most likely consistent with model M13 (both processes are equally affected by such modulation), although model M14 also provides a noteworthy alternative (only the fast process is affected by this modulation).

## Discussion

We examined the coupling of adaptation between reciprocal rotations of a hammer-like tool. Two groups of subjects performed the task. For the first group, the visual information about the object’s configuration was provided and was informative of the object’s dynamics (Explicit group). For the second group, the visual information was ambiguous. We developed a context-dependent state-space model to evaluate coupling for each group, where coupling was considered as the extent to which an error experienced for one rotational direction affected the adaptive behaviour for the other. Our results show that whether the visual configuration of the object was visually explicit or ambiguous, there was substantial coupling of adaptation when manipulating the object across the different directions of rotation. Moreover, providing visual information about the configuration of the object strongly enhanced this coupling, such that learning generalized further between contexts.

Our results are in accord with previous studies suggesting that during manipulation of a single object, separate representations are required to account for adaptation in each context^4^. In the current study, models that assumed a single representation for the object (i.e. full coupling between movement direction; M1 & M4) have very poor performance compared to models with no or intermediate coupling. The ability of the motor system to form separate context-dependent representations for a single object may be a fundamental feature of object manipulation^4,16,17,26^. This may be beneficial particularly when the dynamic behaviour of an object changes from one context to another. For example, when using a handsaw or rowing with an oar, the required load force depends entirely on the direction of each movement back and forth. The extent to which these context-dependent representations interact (e.g., via coupling), seems to depend on the nature of the context they are associated with^27^, or the dynamics experienced in each context^5^. For example, it has been suggested that when an object has the same dynamic behaviour in separate contexts, a unified representation is formed for the object across these contexts^5^ (equivalent to full coupling between the contexts). Whereas, when the dynamics differs from one context to another, separate representations account for the adaptation in each context^4,5^. Our results suggest that the visual configuration of an object further determines the interaction (coupling) between these context-dependent representations, even when the dynamic properties of the object are the same and visual error feedback is always provided. As such, when the visual configuration is explicit, the formed representation is more cohesive, and generalizes across contexts to a greater extent (stronger coupling). Whereas, an ambiguous visual configuration, which is not, in advance, informative of the dynamics, leads to the formation of a representation that is more context specific.

The transfer of learning across movement directions has been studied in other dynamic tasks such as force field adaptation. In these tasks, subjects adapt to novel dynamics during reaching movements, while no visual information is provided to cue changes in the dynamics. It has been shown that the generalization of learning in such tasks is limited, with movements in opposite directions show no or minimal coupling of adaptation^28–31^. In these tasks, knowledge about the dynamics comes from the physical interaction, while visual feedback does not contribute to this knowledge. Similarly, in our task with ambiguous visual information, subjects experienced the dynamics only through physical interaction, which also led to limited coupling of adaptation. When visual information about the object’s configuration was provided, coupling was enhanced by more than twofold (i.e., from 12% to 31% based on M13; Table 2). This suggests that the visual information contributes more to the coupling of adaptation across contexts than physical interaction alone.

The effect of visual configuration on generalization may reflect the differences in structural versus parameter learning. For example, when manipulating an object, the representation of its dynamics consists of knowledge of the dynamic structure (i.e., state-dependency of forces and torques) as well as the knowledge of the parameters of the dynamics (i.e., the mass, moment of inertia, etc)^32^. The coupling of adaptation might, therefore, be due to both the generalization of the dynamic structure, and the generalization of parameterization (learning the parameters of the dynamics). It has been suggested that the dynamic structure of a tool generalizes more broadly across contexts, while parameterization generalizes only to a limited extent^4^. For example, when the orientation of an object with respect to the grasp point changes, subjects generate forces in the correct direction for the new orientation, but with a magnitude that decreases as the grasped orientation varies for the trained orientation^4^. This suggests that the object’s visual configuration is informative with regard to the structure of the task in all orientations, leading to applied forces in the correct directions, but the learned parameters of the task (e.g., expected mass) which contribute to the magnitude of the force only transfer narrowly across changes in orientation. Therefore, in our task when the visual configuration of an object is provided (Explicit group), it informs the structure of the dynamics, which is shared (coupled) across both rotational directions. This shared information, therefore, contributes significantly to the observed coupling. By contrast, when the visual configuration of the object is ambiguous, it is not informative of the dynamic structure and how it might vary between the contexts. Therefore, the contribution of the dynamic structure to coupling is substantially reduced.

From a different perspective, two sources of information might contribute to the formation of our representation about an object: the sensory prediction error, provided from visual and haptic (proprioceptive) feedback during tool use, and the prior knowledge about the dynamics of similar objects used in the past. When the visual configuration is provided and it is of a familiar shape and configuration, it retrieves the prior knowledge about similar objects used in the past and how they might behave in different contexts. For a hammer-like object, for example, we expect a consistent dynamics in different rotational directions, meaning that the Out and Return rotations are expected to show the same dynamics (fully coupled). On the other hand, the sensory prediction error shows that the dynamics might vary from one rotational direction to the other, thus the coupling should be reduced. The integration of these two sources of information leads to an intermediate coupling between Out and Return rotations. When the visual configuration is ambiguous, however, our representation of the object relies mostly on the sensory prediction error, which encourages the motor system to form separate representations for each rotational direction, leading to reduced coupling.

In order to ensure that coupling was accounted for in all possible pairs of trial types, several blocks of trials were used in the experimental design in which the dynamics varied in different ways between Out and Return rotations. The order of the blocks, in this case, was fixed across subjects and groups to allow model fitting to the average data, instead of noisy individual data, and to better contrast the effect of visual configuration on coupling while controlling for the history of adaptation across groups. We acknowledge that having a fixed order of blocks across subjects might obscure any changes in coupling across the course of the experiment. However, we argue that this does not affect the conclusions of our study. Our main focus here was to examine how the coupling differed between groups as a function of visual configuration, rather than changes in coupling across the blocks within each group. In this case, a single coupling factor accounts for the overall coupling throughout the paradigm and is compared between the groups. Indeed, further work is required to assess the possible mechanisms that underlie coupling as a function of adaptation history and how it might change across different blocks.

Our results suggest that the way people generalize dynamic learning of a tool relies not only on its dynamic behaviour, but also on the visual configuration with which the dynamics is associated. Moreover, in our task the visual information substantially increases generalization (coupling) relative to physical interaction alone. This suggests that contextual information, such as the visual configuration, may be a greater determinant of generalization than physical interaction.

## Methods

The study was approved by the Cambridge Psychology Research Ethics Committee, and all methods were performed in accordance with the relevant guidelines and regulations. Twenty-four healthy right handed subjects (14 female; age between 20 and 30 years) provided informed written consent before participating in this study. All subjects were naive to the purpose of the experiment. Subjects sat at a virtual reality system and rotated a virtual hammer-like object in the horizontal plane by grasping and rotating the vertical handle of a robotic manipulandum (the WristBOT^20^; Fig. 1A). The WristBOT simulated the dynamic behaviour of the object during rotation, generating both translational forces in the horizontal plane and a torque about the vertical handle. Visual feedback associated with the object and task was provided in real time using a virtual reality display system (horizontal monitor and mirror). The latency of the display was on average 8 ms, measured with a photosensor on the monitor, plus 0.5 ms latency to read the robot encoders (due to the frequency of the robot’s control loop, 2000 Hz). The visual object consisted of a circular handle (radius 0.5 cm) attached to a 4 cm square mass by an 8 cm rod (width 0.2 cm; Fig. 1B). The position and orientation of the object were determined by the position and orientation of the WristBOT handle.

### Task

The task was to rotate the object back and forth between two angular targets while keeping the position of the handle stationary at the centre of the home region (a visually presented 1 cm radius disc; Fig. 1A). This required subjects to generate rotational movement about the object’s handle and counteract any translational forces. The trials consisted of alternating clockwise (CW) and counter-clockwise (CCW) rotations between the targets (oriented bars 40° apart emanating from the home region; Fig. 1B). Each trial began with the handle of the object stationary within the home region and the rod of the object aligned with either the CCW target or the CW target. Movement initiation was cued by a tone and the appearance of the other target, towards which subjects rotated the object. The trial ended when the orientation of the object was aligned with the orientation of the target. Subjects were required to finish the movement within 400 ms, otherwise they were warned with a ‘two slow’ message. Movements exceeding 500 ms had to be repeated.

Previous studies have shown that a current movement plan could be affected by its preceding^33^ or following^34,35^ actions, when such actions occur within a time interval prior or after the current movement (i.e., 0–500 ms). Such an effect diminishes as the delay between sequential movements increases, such that for delays larger than 600 ms the effect is removed^33^. To avoid such effects between CW and CCW rotations in our task, an inter-trial delay of 750 ms was introduced. In addition, each trial required subjects to remain stationary at the home/target position for 100 ms both before the movement onset and after the movement was finished. This provided at least 950 ms time delay between the CW and CCW rotations, making these movements clearly separate.

The dynamics of the object was simulated as a point mass (1% of the subject’s body mass) at the end of the rigid rod (Fig. 1B). Rotating the object generated forces and torques at the handle which were simulated by the manipulandum. The torque was associated with the moment of inertia of the object, and the force was associated with the circular motion of the mass (Fig. 1B). The direction of force depended on the orientation of the object, while its magnitude was determined by the mass and the length of the rod^21^. Critically, the force caused the handle of the object to displace unless subjects produced a compensatory force in the opposite direction.

During the experiments, we manipulated the dynamics of the object according to one of three possible trial types: exposure trials, zero-force trials or error-clamp trials (Fig. 1C). On exposure trials, subjects experienced the full dynamics of the object including the force on the handle as well as the torque. In these trials, subjects had to learn to compensate for the translational forces in order to keep the handle stationary during the rotation. On zero-force trials, the manipulandum did not produce any translational forces and the handle was free to move. Importantly, any forces produced by subjects on zero-force trials would cause the handle to displace. Finally, on error-clamp trials, the manipulandum simulated a stiff two-dimensional spring, centred on the handle position at the start of the trial (the spring constant was 40 N/cm). Error-clamp trials effectively eliminated kinematic errors and prevented error-driven adaptation. They also allowed the compensatory forces produced by subjects to be measured^21^. On all trial types, the torques associated with the moment of inertia of the object were generated.

### Analysis

We measured the position and orientation of the handle at 1000 Hz and for each trial we extracted only those samples where the angular velocity of the handle rotation exceeded 5% of its peak value for the trial. We calculated the displacement of the handle relative to the first time point for each trial.

Two performance measures were calculated. On exposure and zero-force trials, we calculated the kinematic error (KE) and on error-clamp trials we calculated the adaptation index (see below). Because of the direction of the force at the handle, produced by CW and CCW rotations of the object, the kinematic errors will tend to align with a single axis. Therefore, to produce a signed error we projected each subjects handle position onto the axis of the first principal component computed over all zero-force and exposure trials. On average the first principal component accounted for 86.5 ± 4.5% (mean ± std) of the variance in handle position and the angle on the principal components was 10.2° ± 4° (circular mean ± std from the horizontal line). As a measure of KE we calculated the mean of the projected position across the trial onto the first principal axis. As most trials had a dominant positional displacement on this axis, this measure captures performance. We used a sign convention so that on exposure trials the KE would be positive for under-compensation and that in zero-force trials an aftereffect would be negative.

On error-clamp trials, we measured the time course of the force magnitude generated by subjects and also calculated the ideal force trajectory that would fully compensate for the object dynamics based on the angular velocity and acceleration on that trial (that is, Fig. 1B). As with KE, we included only those samples where the angular velocity exceeded 5% of its peak value for the rotation. We then regressed the measured force onto the ideal force (without intercept) and took the regression coefficient as the *adaptation index*, with 0 representing no adaptation and 1 full adaptation.

To evaluate the learning rate for adaptation in block 12 (Fig. 4C and D), we fit a simple state-space model with one retention factor and one learning rate to the data: ${z}^{(n+1)}=A\cdot {z}^{(n)}+B\cdot (1-{z}^{(n)})$, where *A* and *B* are the retention and learning rate parameters, respectively, and ${z}^{(1)}={z}_{0}$ is the initial value of adaptation (as a free parameter). For adaptation in the Out movements, we fit $1-{z}^{(n)}$ to the KE data for each group separately. Similarly for the Return movements, we fit *z*^(*n*)^ to the adaptation index for each group. The confidence intervals on the free parameters and the *p*-values shown in Fig. 4C and D are obtained from bootstrapping (see below).

### Experimental paradigm

We examined the representation of the object’s dynamics during reciprocal object rotations. Specifically, we asked whether the dynamics of CW and CCW rotations are represented by separate adaptive mechanisms, or whether these mechanisms interact. The experiment included CW and CCW rotations back and forth between two angular targets (Fig. 1A). The first trial of each pair was considered as the “Out” rotation, followed by the second trial as the “Return” rotation (these were discrete trials). We counterbalanced the choice of Out and Return rotations as CW or CCW across the subjects. The data from these subgroups was appropriately combined for the analysis. Each subject had a familiarization session consisting of 20 pairs of zero-force trials. These trials were not analyzed.

Two groups of subjects (n = 12 in each) participated in the experiment. In the first group, the visual information about the configuration of the object was available, while in the second group, we made the configuration ambiguous (Fig. 2A). The first group, therefore, benefited from visual information as to the dynamics of the object, while for the second group, the visual information prior to movement was uninformative as to the dynamics. We examined how visual information about the object’s configuration affects the coupling of adaptation between different movement contexts (that is, Out and Return rotations).

The experiment consisted of various combinations of task dynamics (trial types) between Out and Return rotations. Specifically, subjects performed 13 consecutive blocks of trials, each consisting of 13 pairs of Out-Return rotations (Fig. 2B). Within each block, the dynamics between Out and Return rotations could be different. In some blocks, we applied the same dynamics for both rotations, while in the other blocks, we applied different dynamics for each rotation (Fig. 2B). Each block was preceded by two pairs of error-clamp trials in which we measured the adaptation index at the beginning of that block for each rotation direction (as shown by narrow rectangles in Fig. 2B).

In the first block subjects performed 13 pairs of zero-force trials as the baseline. Subsequently, in blocks 2 to 10, we alternated blocks of concurrent exposure for both rotations (i.e., even-numbered blocks) with blocks in which we varied the dynamics between the two rotations (i.e., odd-numbered blocks). We examined all possible combinations of dynamics in these blocks in which the Return movement was a clamp trial, while the Out movement was either a clamp (block 3), exposure (block 5), or zero-force (block 7). We also examined cases where the Out movement was a zero-force trial and the Return movement was either a clamp (block 7) or exposure (block 9) trial. In block 11, subjects had a washout period (zero-force trials) for both rotations to return their performance back to the baseline. We then examined learning after washout in block 12 in which the Out movement was an exposure and Return movement was a clamp trial. Finally, subjects finished the experiment with a zero-force (washout) block for both movement rotations in block 13.

All subjects underwent the same order of blocks so that the behaviour in each block was comparable across subjects. This allowed us to average the time course of adaptation index and KE across all subjects, and perform model fitting on the averaged data over the whole trial sequence.

Subjects received short rest breaks (45 seconds) at the end of blocks 4, 8 and 11. After each rest break, subjects performed 5 additional pairs of trials of the same block to restore their performance level back to the level prior to the rest break. These additional trials were excluded from the analysis.

### Model fitting

For each group, we fit models (see main text for details) to the adaptation index and the kinematic error (KE), simultaneously. To this end, the net motor output in the model was fit to the adaptation index, and the error term was fit to the KE. We used four mapping parameters to match the adaptation index and KE values in the experimental data with the equivalent data from the model (i.e., the net output and the error) using a linear mapping (each performance measure in the data was independently mapped to its corresponding model variable via a scaling and an offset parameter^22^). The Bayesian information criterion (*BIC*) was used to select the best model for each group (equation 5):

5 $$BIC=N\cdot \,\mathrm{ln}(\frac{SSE}{N})+p\cdot \,\mathrm{ln}(N)$$

where, *N* is the number of data points for both groups put together, *SSE* is the sum of squared errors of the model fit, and *p* is the degrees of freedom of the model. The relative *BIC* value (i.e., Δ*BIC* in Table 1) between two models represents half the log of the Bayes factor^36^. A Δ*BIC* of greater than 4.6 (a Bayes factor of greater than 10) is considered to provide strong evidence in favour of the model with the lower *BIC*^37^. After fitting all models to the average KE and adaptation index, we calculated the difference in *BIC* between each model and the preferred model (with the smallest *BIC*). This provided a Δ*BIC* value for each model (with 0 for the selected model) as shown in Table 1.

In order to obtain the confidence intervals over the model parameters we performed a bootstrapping analysis^38^. For each group of subjects (n = 12), we randomly selected 12 subjects (with replacement), and fit the models to the mean of each of 10,000 such bootstrap samples. The 95% confidence interval over each parameter for a given model was obtained based on the 2.5th and 97.5th percentile of the parameter distribution across all samples.

The statistical test on the comparison of parameter values across groups (i.e., Figs 4C and D and 6) was performed by taking the difference between the bootstrap distributions corresponding to each parameter (i.e., the difference between all possible samples), and performing a two-tailed test on the resultant distribution against zero.

## Data Availability

The authors are happy to make the data used in this study available upon request.
